# The Role of Left-Atrial Mechanics Assessed by Two-Dimensional Speckle-Tracking Echocardiography to Differentiate Hypertrophic Cardiomyopathy from Hypertensive Left-Ventricular Hypertrophy

**DOI:** 10.3390/diagnostics11050814

**Published:** 2021-04-30

**Authors:** Nicoleta-Monica Popa-Fotea, Miruna Mihaela Micheu, Nicoleta Oprescu, Adriana Alexandrescu, Maria Greavu, Sebastian Onciul, Roxana Onut, Ioana Petre, Alina Scarlatescu, Monica Stoian, Razvan Ticulescu, Diana Zamfir, Maria Dorobanțu

**Affiliations:** 1 Cardio-thoracic Department, University of Medicine and Pharmacy Carol Davila, Eroii Sanitari Bvd. 8, 050474 Bucharest, Romania; fotea.nicoleta@yahoo.com (N.-M.P.-F.); sebastian.onciul@gmail.com (S.O.); i_comanescu@yahoo.com (I.P.); maria.dorobantu@gmail.com (M.D.); 2 Department of Cardiology, Clinical Emergency Hospital of Bucharest, Calea Floreasca 8, 014461 Bucharest, Romania; nicoleta_m_oprescu@yahoo.com (N.O.); adrianaurgenta@yahoo.com (A.A.); onutroxana@yahoo.com (R.O.); alina.scarlatescu@gmail.com (A.S.); monica.predescu@gmail.com (M.S.); diana_zam74@yahoo.com (D.Z.); 3 Cardiomyopathy Center, Monza Hospital, Tony Bulandra Street, No. 27, 021967 Bucharest, Romania; r0zmaria@yahoo.com (M.G.); razvanticulescu@gmail.com (R.T.)

**Keywords:** hypertrophic cardiomyopathy, left-ventricular hypertrophy, left-atrial function, speckle-tracking echocardiography, sarcomeric genes, sarcomeric-associated genes

## Abstract

Hypertrophic cardiomyopathy (HCM) and arterial hypertension (HTN) are conditions with different pathophysiology, but both can result in left-ventricular hypertrophy (LVH). The role of left-atrial (LA) functional changes detected by two-dimensional speckle-tracking echocardiography (STE) in indicating LVH etiology is unknown. Methods: We aimed to characterize LA mechanics using STE in LVH patients with HCM and HTN. LA 2D volumetric and STE parameters were analyzed in 86 LVH patients (43 HCM and 43 isolated HTN subjects) and 33 age- and sex-matched controls. Results: The volumetric study showed that LA reservoir and conduit function were impaired in the HCM group compared to controls, while, in the HTN group, only LA conduit function was deteriorated. The HCM group had all three STE-derived LA functions impaired compared to controls. The HTN group, consistently with volumetric analysis, had solely LA conduit function reduced compared to controls. Ratios of LA booster-pump strain (S) and strain rate (SR) to interventricular septum (IVS) thickness were the most accurate parameters to discriminate between HCM and HTN. The subgroup harboring sarcomeric pathogenic (P)/likely pathogenic (LP) variants had reduced LA booster-pump S and SR compared with the genotype-negative subgroup. Conclusions: LA reservoir, conduit, and pump functions are decreased in HCM compared to HTN patients with similar LVH. We report the ratios between LA contraction S/SR and IVS thickness as novel parameters with high accuracy in discriminating LVH due to HCM. The presence of P/LP variants in sarcomeric or sarcomeric-associated genes could be associated with more severe LA dysfunction.

## 1. Introduction

Hypertrophic cardiomyopathy (HCM) is an autosomal dominant disease caused by mutations in genes encoding sarcomeric or sarcomere-associated proteins [1]. Left-ventricular hypertrophy (LVH) in HCM develops through complex mechanisms involving contractile dysfunction of both atria and ventricles due to impaired actin–myosin interaction [2,3], while, in arterial hypertension (HTN), LVH is related to increased pressure afterload [4]. The clinical phenotype differentiation of the two diseases is frequently challenging, mainly because the penetrance of LVH in HCM is age-dependent, and the prevalence of HTN also increases gradually with age. 

The contribution of the left atrium (LA) to cardiovascular performance in HCM patients is getting increased recognition, in terms of both size and function. LA maximal and minimal volumes are robust surrogate biomarkers of LV diastolic dysfunction [5,6], while LA function correlates with exercise capacity [7] and heart failure in HCM [8]. LA and LV functions are closely interrelated throughout the cardiac cycle. The primary role of LA is to modulate LV filling by working as a reservoir for pulmonary venous return during ventricular systole, a conduit for pulmonary venous return during early ventricular diastole, and a booster-pump during late ventricular diastole [9].

The assessment of LA mechanics by two-dimensional (2D) speckle-tracking echocardiography (STE) is already a widely accessible technique with feasible parameters for LA function [10,11]. Changes in LV function are well studied in both HCM and HTN, but LA remodeling is not yet completely understood. 

The main goal of this study was to assess the phasic function of LA in a well-characterized genotyped cohort of HCM probands compared to a hypertensive LVH group. 

## 2. Materials and Methods

### 2.1. Study Population

The study was approved by the Ethics Committee of the Clinical Emergency Hospital of Bucharest (approval no. 2067, 26 February 2017and performed in compliance with the principles of the Declaration of Helsinki). 

The study population comprised unrelated index patients with HCM that fulfilled the diagnostic criteria of the European Society of Cardiology [12] (HCM group). We also included subjects with hypertrophic LV remodeling induced by isolated HTN, defined as a repeatedly measured systolic blood pressure ≥140 mm Hg or diastolic blood pressure ≥90 mm Hg or receiving optimal antihypertensive pharmacotherapy in the absence of valvular or metabolic disorders that could induce LV hypertrophy (HTN group). The subjects from both groups were screened at two centers, Emergency Clinical Hospital and Monza Hospital, between 2017 and 2020; the subjects screened at Monza Hospital were further referred to Emergency Clinical Hospital for the entire study protocol. We excluded from both cohorts, HCM and HTN, the subjects with current or prior atrial fibrillation (AF), inadequate acoustic window, LVH of other causes (such as myocardial storage or valvular diseases), and pulmonary hypertension (defined as a noninvasive pulmonary artery systolic pressure >45 mmHg). We chose to compare HCM with the hypertension LVH group as it is frequently encountered and represents an LVH model. 

The control group consisted of age- and sex-matched healthy individuals free of cardiovascular risk factors, with normal physical examination, electrocardiogram, echocardiography, and arterial blood pressure, presenting at our center for routine medical examinations. All subjects underwent clinical work-up, including medical history, physical examination, 12-lead electrocardiogram, and two-dimensional transthoracic echocardiography (2D-TTE), as previously reported [13]. 

### 2.2. Genetic Testing

Patients in the HCM group underwent genetic testing following a methodology that was described in detail elsewhere [14,15]. In summary, blood samples were collected at enrollment, and DNA was isolated using the MagCore Genomic DNA Whole Blood Kit (RBC Bioscience, Taipei, Taiwan). Targeted sequencing was performed on an Illumina MiSeq platform using TruSight Cardio Sequencing Kit (Illumina, San Diego, California, SDSU, USA) following the manufacturer’s instructions.

The procedure targeted 47 core and emerging genes associated with HCM, including genes encoding for sarcomere and sarcomere-associated proteins. Variants were classified according to currently available standards and guidelines, taking into account evidence such as allele frequency in control populations, genotype segregation, amino-acid conservation, and in silico prediction [16].

### 2.3. Echocardiographic Image Acquisition

All subjects underwent a comprehensive 2D-TTE and Doppler study using VIVID E9 (GE Healthcare, Wauwatosa, WI, USA) with a 3.5 MHz array probe in standard left lateral position. A complete 2D-TTE dataset was acquired including dedicated apical two- and four-chamber views, avoiding LA foreshortening with a frame rate between 50 and 70 frames/s and a record of at least three heartbeats for LA strain assessment. The datasets were analyzed offline on EchoPac, version BT13 (GE Vingmed Ultrasound, Horten, Norway).

#### 2.3.1. Conventional Echocardiography Analysis

Conventional echocardiographic measurements were performed according to current guidelines for chamber quantification [17]. Left-ventricular ejection fraction (LVEF) was calculated using Simpson’s biplane method and LA diameter was assessed by 2D-TTE in parasternal long-axis view in end-systole. Diastolic function was analyzed taking into account mitral E and A wave velocities and E/A ratio, as well as early-diastolic septal (e’s) and lateral (e’l) mitral annular velocities using tissue Doppler imaging [5]; then, the average e’m was calculated as (e’l + e’s)/2. Cardiac output was assessed as the heart rate multiplied by stroke volume, the latter being the result of LV outflow tract (LVOT) area multiplied by the LVOT velocity time integral. Cardiac index was calculated as cardiac output divided by body mass area. The mitral annulus diastolic diameter was measured in the apical four-chamber view, and the mitral annulus area was then derived [18]. Subsequently, LA ejection force (LAEF) was calculated using the following formula: 0.5 × 1.06 × mitral annulus area × (peak A velocity^2^) and expressed as kdyne [19].

#### 2.3.2. Left Atrial 2D Volumetric Analysis

Two-dimensional phasic LA volumes were calculated as the average of the volumes measured in apical two- and four-chamber views using the disc summation algorithm. Maximal volume (LAV_max_) was measured just before mitral valve opening, LA minimum volume (LAV_min_) was measured at mitral valve closure, and LA pre-A-wave volume (LAV_pre-A_) was measured one frame before atrial contraction in accordance with American Society of Echocardiography and European Association of Cardiovascular Imaging guidelines for chamber quantification [20]. Using the above volumes, LA total (LATEV), passive (LAPEV), and active emptying volumes (LAAEV) and fractions (LATEF, LAPEF, LAEI, and LAAEF) were derived [20], as shown in Table S1 (Supplementary Materials). All volumes were indexed to body surface area.

#### 2.3.3. LA 2D Speckle-Tracking Analysis

LA longitudinal strain (LAS) and strain rate (LASR) were assessed using 2D-STE, with the values being averaged over the apical four- and two-chamber views. The endocardial border of LA was manually traced such that the region of interest followed the LA endocardial border throughout the cardiac cycle. Time–strain and time–strain rate plots were automatically created by the software. The time–strain plot was used for assessing the following parameters: LA strain reservoir (LASr) representing the difference between the strain value at mitral valve opening and left-ventricular end-diastole (LVED), LA conduit strain (LAScd) representing the difference between strain at onset of atrial contraction and mitral valve opening, and LA contractile strain (LASct) representing the difference between strain value at LVED and onset of atrial contraction [21]. A zero-strain reference was set at LVED. The time–strain rate plot was used to measure the following parameters: LA reservoir strain rate (SR) (pLASRr) as the peak systolic positive value, LA conduit SR (pLASRcd) as the early diastolic negative peak, and LA contractile SR (pLASRct) as the late diastolic negative peak. Each of the STE-derived LA parameters was normalized to the interventricular septum (IVS) thickness (LA strain/strain rate to IVS ratios). The LA stiffness index (LASI) was defined as E/e′m divided by LASr [22,23].

### 2.4. Reproducibility Assessment

For the assessment of inter-observer variability, two independent investigators (N.-M.P.-F. and N.O.) performed the offline analysis related to LA function in a subgroup of 30 random patients included in the study. To determine the intra-observer variability, one of the investigators (N.-M.P.-F.) reanalyzed LA mechanics in a random order with a minimum of 7 days between two corresponding assessments.

### 2.5. Statistical Analysis

All analyses were conducted using the statistical software program SPSS version 23 (IBM, Armonk, NY, USA). Data were presented as the mean ± SD for continuous variables and as the number and percentage for categorical variables. For normally distributed variables, differences between groups were compared with Student’s *t*-test or chi-square test. Binary logistic regression analysis was performed to assess the variables able to differentiate LVH due to either HCM or HTN after adjustment for age and sex; independent variables were selected on the basis of their relevance in the baseline tables. Variables with *p* < 0.05 in univariable analysis were selected for the multivariable model. To evaluate the discriminative power of LA parameters between HCM and HTN, receiver operating characteristic curve (ROC) analysis was performed to determine the area under the curve (AUC) and optimal sensitivity and specificity. Intra- and inter-observer agreements for LA parameters were calculated with intraclass correlation coefficients. The *p*-values were two-tailed, and a cutoff of less than 0.05 was considered statistically significant. 

## 3. Results

### 3.1. Study Population and Conventional Echocardiography Parameters

Fifty-four subjects were initially screened for inclusion in the HCM group; however, 11 were eventually excluded, six due to episodes of paroxysmal AF and five because of an inadequate acoustic window. In the HTN group, 53 subjects were initially screened; however, 10 were eventually excluded due to a poor echographic window. The demographic and conventional echocardiography parameters of the subjects included are summarized in Table 1: 43 subjects with HCM, 43 subjects with HTN, and 33 controls. The mean age of the HCM group was 50 ± 14.5 years old, and around 78% were males. There were no significant differences regarding age or sex among the three groups. Concerning conventional echocardiography, LVEF, LVEDD, LVESD, LVEDV, and LVESV did not differ across the three groups (Table 1). Cardiac index was significantly decreased solely in the HCM group (3.2 ± 0.38 L/min/m^2^) compared with the control (3.5 ± 0.5 L/min/m^2^). Interventricular septum (20.24 ± 4.32 mm) and indexed LV mass (131.85 ± 44.77 g/m^2^) were significantly increased in the HCM group compared with the HTN group (16.2 ± 2.6 mm and 92 ± 30.9 g/m^2^, respectively) or control (8.2 ± 1.7 mm and 71 ± 19.23 g/m^2^, respectively). 

### 3.2. Reproducibility Analysis

All LA deformation parameters showed good to excellent reproducibility with intraclass correlation coefficients of 0.71 to 0.94 (Table S2, Supplementary Materials). 

### 3.3. LA Volumetric Analysis 

The analysis of LA dimensions indicated that all three measures of LAV were numerically larger in the HCM group compared to controls, while, in the HTN group, only LAV_pre-A_ (21 ± 1.4 mL/m^2^) was significantly larger in contrast to healthy controls (*p* = 0.01) (Table 2). Using volumetric analysis, in the HCM group, LA reservoir and conduit functions were depressed, as shown by the reduced LATEF (55% ± 9.2%), LAEI (168% ± 50.5%), and LAPEF (25% ± 10.5%) compared with healthy controls (60% ± 10.3%, 200% ± 87.2%, and 31% ± 11.2%, respectively). In HTN group, only the LA conduit function was reduced compared with the control, with LAPEF calculated at 26% ± 9.1%, *p* = 0.035. LA booster-pump ratios evaluated with the parameters LAAEF and LAEF were similar to those of the controls in both HCM and HTN groups, with no differences within groups. 

### 3.4. LA 2D Speckle-Tracking Analysis

**Figure 1:**
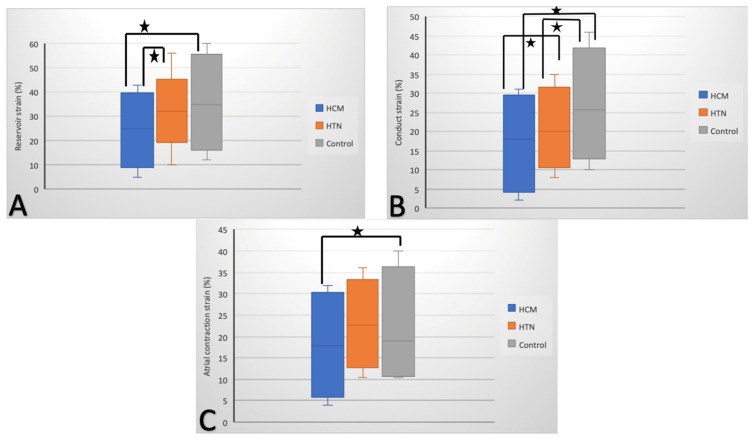
Left-atrial reservoir (**A**), conduit (**B**), and atrial contraction (**B**) strain (%) in patients with hypertrophic cardiomyopathy (HCM), patients with hypertension (HTN), and controls; * *p* < 0.05 for HCM–control and HCM–HTN comparisons (**A**), for HCM–control, HCM–HTN, and HTN–control comparisons (**B**), and for HCM–control comparisons (**C**).

According to 2D-STE, in the HCM group, all three LA strain and strain rate measurements were impaired compared to controls: reservoir (S 24% ± 5.8%, SR 1 ± 0.89), conduit (S 18% ± 7.1%, SR −0.5 ± 0.1), and booster-pump (S 11% ± 2.1%, SR −1.4 ± 0.3) (Figure 1). In the HTN group, only LA conduit function was reduced, with LA strain (22% ± 1.3%) and strain rate (−1.2 ± 1.3 s^−1^) being decreased compared to controls (25% ± 4.6% and −1.8 ± 0.95 s^−1^, respectively), but not to the same extent as in the HCM group (Figure 1b). LA reservoir and conduit functions were significantly decreased in the HCM group compared with the HTN group, while the difference within groups concerning LA booster-pump function was at the limit of significance (Table 2). 

### 3.5. The Novel Strain-To-Thickness Ratio

Ratios of LA S and SR to IVS thickness for each LA function were reduced in the HCM group compared to the HTN group. The multivariate logistic regression analysis indicated only LASct/IVS and pLASRct/IVS as significant discriminators differentiating LVH due to HCM (Table S3, Supplementary Materials). 

**Figure 2:**
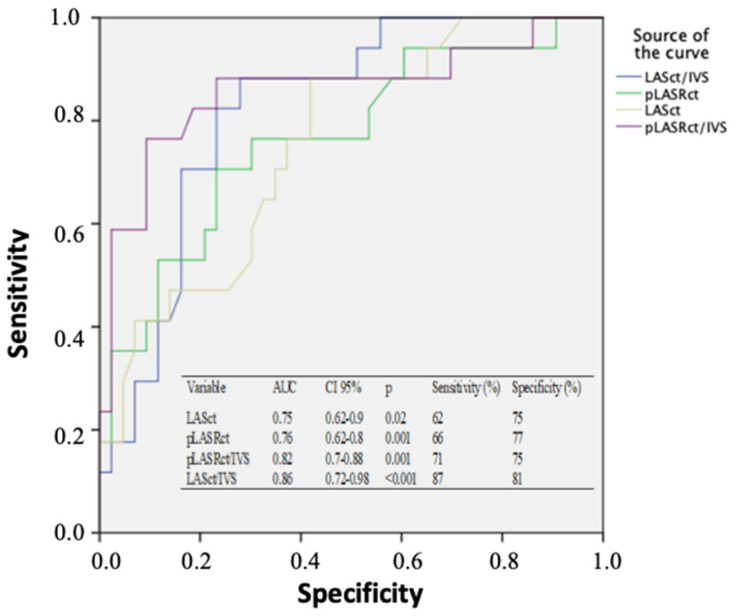
Receiver operating characteristic curves and the corresponding data for left-atrial mechanical parameters to differentiate hypertrophic cardiomyopathy from arterial hypertension with left-ventricular hypertrophy. AUC, area under the curve; CI, confidence interval; IVS, interventricular septum; LASct, left-atrial strain during contractile phase; pLASTct peak strain rate during contractile phase.

To explore the cutoffs that discriminate HCM from HTN with LVH, depending on LA deformation, ROC curves were calculated (Figure 2). Only atrial contractile function had extrapolative values; LASct/IVS and pLASRct/IVS showed the best AUC as follows: LASct/IVS ≤ 0.38 showed 87% sensitivity and 81% specificity, AUC 0.86 (CI: 0.72–0.98, *p* < 0.001); pLASRct/IVS ≤ −0.03 showed 71% sensitivity and 75% specificity, AUC 0.82 (CI: 0.7–0.88, *p* = 0.001). For conduit and reservoir functions, the AUC was very small (<0.3), and no cutoff points could be obtained to differentiate HCM from HTN.

### 3.6. LA Mechanics According to Genotype

The group with HCM and pathogenic (P)/likely pathogenic (LP) variants had similar indexed LV mass, LVEF, and LA diameter. All phasic volumes were comparable between HCM groups with the exception of LAV_pre-A_, which was slightly small in those with P/LP variants compared with genotype-negative or HTN subjects. Volumetric analysis of LA function did not display differences within the HCM group according to genotype. However, in STE, after dividing the HCM group according to the presence of P/LP variants in sarcomeric proteins, HCM subjects with P/LP variants had lower LA contractile S (10.3% ± 1.9%) and SR (−1.15 ± 0.4 s^−1^) compared to HCM subjects without P/LP variants (11.4% ± 2.37% and −1.5 ± 0.23 s^−1^, respectively) (Table 3).

## 4. Discussion

This study examined LA function using conventional 2D volumetric and STE-derived parameters in subjects with LVH due to either HCM or HTN, as well as analyzed whether P/LP variants had an impact on LA functional remodeling. Speckle-tracking echocardiography proved to be a reproductible technique [24,25], easy to perform in routine practice in subjects with an appropriate acoustic window. Our results show that LA function as assessed by 2D-STE is globally more affected in HCM compared to HTN subjects, with the latter group being characterized by isolated LA conduit dysfunction, while reservoir and booster-pump functions were preserved; these findings are similar to those in other studies reporting a decreased pLASRcd in HTN [26]. On the other hand, the two-dimensional volumetric analysis showed that HCM subjects displayed only a reduction in the LA reservoir and conduit functions, while LA booster-pump function was similar to the control and HTN groups. These differences between volumetric and STE analysis may be explained by a higher sensitivity of STE in revealing subclinical myocardial dysfunction. The reduction in LA passive filling in HCM evaluated through reservoir and conduit function is most likely due to the reduction in LA compliance [27], evaluated indirectly through LASI, which showed significantly increased stiffness in HCM subjects compared with controls and the HTN subgroup (as detailed in Table 3). 

As a novelty, we propose, for the first time, as far as we know, the normalization of the LA strain parameters to IVS thickness. We show that ratios of LA strain and strain rate to IVS thickness are better discriminators of HCM. Precisely, the LASct-to-IVS ratio demonstrated high sensitivity (87%) and specificity (81%) for a cutoff of ≤0.38, as did the pLASRct-to-IVS ratio with 71% sensitivity and 75% specificity for a cutoff of ≤−0.03, compared with other classic echocardiographic parameters that showed neither sensitivity nor specificity. Consequently, ratios of LASct and pLASRct to IVS may be promising markers for differentiation between HCM and HTN as causes of LVH, but this needs to be confirmed on larger cohorts and on subjects where the differential diagnosis between HTN and HCM is questionable. Distinctly from Badran et al. [10], we obtained significant differences in LA booster-pump between HCM and HTN groups and, furthermore, LASct/IVS and pLASRct/IVS had better discriminative power for HCM compared with the parameters proposed by the above authors. The differences may be due to the dissimilarities in the characteristics of the included subjects; the LVEF and cardiac index of our HCM group were meaningfully decreased compared with Badran’s cohort, suggesting a more advanced stage of HCM disease in our cohort. 

In cardiac magnetic resonance (CMR)-based studies, it has been demonstrated that HCM is characterized by a staging of LA functional abnormalities, which correlates with the extent of LV fibrosis. Accordingly, LA conduit function is reduced in the early phases of the disease (characterized by mild or absent LV fibrosis), while LA contractile dysfunction develops later in the course of the disease when extensive LV fibrosis is documented [28]. Indeed, LA reservoir and conduit dysfunction, along with regional LA deformation (specifically declined function in the antero-roof LA wall) occurs prior to LA enlargement, as recently evidenced by Yang and colleagues [29]. 

In our study, we also encountered some differences in LA mechanics between genotype-positive and -negative HCM subjects. Patients with P/LP sarcomeric or sarcomeric-related mutations had an impaired LA booster-pump function compared to genotype-negative patients, despite similar LVEF, LA volumes, LA reservoir, and conduit functions. Age is an important confounder that we could not correct, which may in part explain the differences, but the exact meaning of these dissimilarities in LA function based on genotype remains uncertain as new emerging genes are discovered. On the basis of current data, we cannot truly say that genotype-positive subjects have significantly worse diastology than their genotype-negative counterparts; thus, additional studies are needed. This difference in LA function may also be due to the potential time-dependency of the development of LA dysfunction with the course of the disease [30] or due to the clinical symptoms, since we noted reduced LA contractility parameters in the few subjects with diastolic heart failure symptoms from our HCM cohort. Longer evolution of the disease is associated with more serious cardiovascular events, as well as LA enlargement and dysfunction [31]. Another possible explanation of the altered booster-pump LA function with normal reservoir and conduit functions may be related to the proteins altered through certain mutations [32]. Moreover, we plan to conduct long-term follow-up of the cohorts and analyze a potential correlation between the values of LA parameters at inclusion and the outcome of subjects at 1 and 5 years. According to the current data, overall management of subjects with HCM is not influenced by genotype analysis, as there are not enough correlations between prognosis and specific mutations.

Our study had several limitations. It included a small number of subjects with HCM, as we excluded those without genetic testing, which may have influenced the statistical results. The cross-sectional design of the study prevented us from drawing any conclusions about the prognostic value of LA mechanics. Future outcome-based studies with a larger cohort of subjects are needed to accurately determine the role of LA S/SR-to-IVS ratios in HCM. Furthermore, we included only subjects with a clear diagnosis of either HCM or HTN, while these novel parameters should also be analyzed on patients that are difficult to differentiate to further investigate if these metrics are also helpful in this circumstance. Another drawback is represented by the lack of CMR data and three-dimensional (3D) TTE in the HCM group. Late gadolinium enhancement (LGE) allows for LA myocardial fibrosis quantification, which could have explained the difference in LA function between genotype-positive and -negative HCM subjects. Similarly, 3D-TTE allows for a more accurate assessment of LA volumes, being more tightly correlated with CMR than 2D-TTE [33].

## 5. Conclusions

A significant alteration of all LA functions was encountered in the HCM group compared to the LVH due to HTN group. We propose the normalization of the LA strain parameters to the interventricular septum thickness, a parameter that may increase the accuracy of discrimination between HCM and HTN with LVH. HCM patients harboring pathogenic or likely pathogenic mutations in genes encoding sarcomeric or sarcomere-associated proteins are characterized by a more severe LA booster-pump dysfunction.

## Figures and Tables

**Table 1:** Comparison of demographic and conventional echocardiographic data among patients with hypertrophic cardiomyopathy (HCM), patients with hypertension (HTN) with left-ventricular hypertrophy (LVH), and controls.

Variable	HCM (*n* = 43)	HTN wit LVH (*n* = 43)	Control (*n* = 33)	*p* HCM vs. Control	*p* HTN vs. Control	*p* HCM vs. HTN
Age (years)	50 ± 14.5	51.4 ± 13.1	47 ± 11.2	0.29	0.10	0.97
Gender (male) (%)	36 (78.3%)	30 (69.6%)	29 (80.5%)	0.35	0.09	0.2
IVS (mm)	20.24 ± 4.32	16.2 ± 2.6	8.2 ± 1.7	<0.001	<0.001	<0.001
Posterior wall (mm)	14.18 ± 3.6	14.2 ± 1.4	7.6 ± 1.6	<0.001	<0.001	0.972
RWT	0.66 ± 0.13	0.96 ± 0.23	0.35 ± 0.1	<0.001	<0.001	<0.001
Indexed LV Mass (g/m^2^)	131.85 ± 44.7	92 ± 30.9	71 ± 19.23	<0.001	<0.001	<0.001
LVEDD (mm)	40.84 ± 7.58	40 ± 11.6	38 ± 4.8	0.053	0.29	0.68
LVESD (mm)	24.51 ± 8.2	25.8 ± 4.7	27 ± 6	0.11	0.33	0.36
LVEDV (mL)	90.2 ± 15	85 ± 9	86 ± 10	0.18	0.65	0.055
LVESV (mL)	40 ± 15.6	37 ± 9	38 ± 11.3	0.53	0.67	0.28
LVEF (%)	53.2 ± 11.87	55 ± 9.2	56 ± 7	0.19	0.58	0.42
HR (bpm)	70 ± 12	72 ± 9	75 ± 10	0.057	0.17	0.38
Cardiac output (L/min)	5.7 ± 0.6	5.9 ± 0.2	6 ± 0.8	0.066	0.43	0.051
Cardiac index (L/min/m^2^)	3.2 ± 0.38	3.3 ± 0.42	3.5 ± 0.5	0.004	0.062	0.25
LA diameter (mm)	42.77 ± 8.91	40 ± 6.1	37.1 ± 6	0.001	0.04	0.097
E (cm/s)	95.4 ± 4.31	86 ± 9.1	90 ± 5.2	<0.001	0.018	<0.001
A (cm/s)	74.4 ± 3.21	81 ± 5.2	75 ± 2	0.96	<0.001	<0.001
E/A (cm/s)	1.23 ± 0.55	1 ± 3.4	1.1 ± 1.7	0.67	0.87	0.66
e’s (cm/s)	6.2 ± 2.4	10 ± 4.5	10 ± 3.2	<0.001	0.98	<0.001
e’l (cm/s)	8.6 ± 4	9 ± 4	11 ± 3.2	<0.001	0.02	0.64
e’m (cm/s)	7.4 ± 3.1	8 ± 4.1	10.5 ± 1.8	<0.001	0.001	0.45
E/e’s	18.65 ± 15.5	10.7 ± 5	9 ± 1.5	<0.001	0.06	<0.001
E/e’l	12.93 ± 8.6	8 ± 2.5	7 ± 2.2	<0.001	0.07	<0.001
E/e’m	15.8 ± 7.6	9 ± 3.4	8 ± 1.4	<0.001	0.08	<0.001

A, atrial velocity of mitral flow; E, early diastolic velocity of mitral flow; e’l, early diastolic velocity of lateral annulus; e’m, average of early diastolic velocity of lateral and mitral annulus; e’s, early diastolic velocity of mitral annulus; HR, heart rate; IVS, interventricular septum; LA, left atrial; LVEDD, left-ventricular end-diastolic diameter; LVEDV, left-ventricular end-diastolic volume; LVEF, left-ventricular ejection fraction; LVESD, left-ventricular end-systolic diameter; LVESV, left-ventricular end-systolic volume; RWT, relative wall thickness.

**Table 2:** Comparison of left-atrial (LA) volumetric and deformation parameters among patients with hypertrophic cardiomyopathy (HCM), patients with hypertension (HTN) with left-ventricular hypertrophy (LVH), and controls.

Variable	HCM (*n* = 43)	HTN (*n* = 43)	Control (*n* = 33)	*p* HCM vs. Control	*p* HTN vs. Control	*p* HCM vs. HTN
Volumetric Variables and LAEF						
LAV_max_ (mL/m^2^)	32.8 ± 24.3	29.89 ± 17.3	25 ± 10.9	<0.001	0.16	0.526
LAV_min_ (mL/m^2^)	18 ± 6.5	16 ± 5	14 ± 8.2	0.025	0.22	0.114
LAV_pre-A_ (mL/ m^2^)	24 ± 7	21 ± 1.4	19.7 ± 2.6	<0.001	0.01	0.008
LATEF (%)	55 ± 9.2	61 ± 8.9	60 ± 10.3	0.03	0.66	0.003
LAEI (%)	168 ± 50.5	191 ± 89	200 ± 87.2	0.048	0.66	0.144
LAPEF (%)	25 ± 10.5	26 ± 9.1	31 ± 11.2	0.019	0.035	0.64
LAAEF (%)	44 ± 6.9	47 ± 7.8	47 ± 15.6	0.31	0.98	0.062
LAEF (kdyne)	12 ± 3.4	13 ± 1.2	14 ± 6.2	0.077	0.3	0.073
Deformation Variables						
Reservoir Function						
LASr (%)	24 ± 5.8	32 ± 9.7	35 ± 7.2	<0.001	0.14	<0.001
LASr/IVS	1.1 ± 0.2	1.9 ± 0.7	5 ± 2.6	<0.001	<0.001	<0.001
pLASRr (s^−1^)	1 ± 0.89	1.9 ± 0.4	2 ± 0.87	<0.001	0.54	<0.001
pLASRr/IVS	0.1 ± 0.09	0.15 ± 0.1	0.3 ± 0.08	<0.001	<0.001	0.017
LASI	1.7 ± 0.5	0.35 ± 0.2	0.24 ± 0.1	<0.001	0.005	<0.001
Conduit Function						
LAScd (%)	18 ± 7.1	22 ± 1.3	25 ± 4.6	<0.001	<0.001	<0.001
LAScd/IVS	1.1 ± 0.7	1.5 ± 0.2	3.6 ± 1.8	<0.001	<0.001	0.001
pLASRcd (s^−1^)	−0.5 ± 0.1	−1.2 ± 1.3	−1.8 ± 0.95	<0.001	0.029	<0.001
pLASRcd/IVS	−0.03 ± 0.1	−0.07 ± 0.03	−0.16 ± 0.1	<0.001	<0.001	0.014
Contractile Function						
LASct (%)	11 ± 2.1	12 ± 2.5	13 ± 2.7	0.001	0.1	0.05
LASct/IVS	0.5 ± 0.1	0.8 ± 0.2	1.87 ± 0.9	<0.001	<0.001	<0.001
pLASRct (s^−1^)	−1.4 ± 0.3	−1.5 ± 1.3	−1.61 ± 0.35	0.006	0.62	0.62
pLASRct/IVS	−0.05 ± 0.1	−0.1 ± 0.09	−0.2 ± 0.1	<0.001	0.001	0.017

LAAEF, left-atrial active ejection fraction; LAEF, left-atrial ejection force; LAEI, left-atrial ejection index; LAPEF, left-atrial passive ejection fraction; LATEF: left-atrial total ejection fraction; LAScd, left-atrial strain during conduit phase; LASct, left-atrial strain during contractile phase; LASI, left-atrial stiffness index; LASr, left-atrial strain during reservoir phase; pLASRr, peak strain rate during reservoir phase; pLASRcd, peak strain rate during conduit phase; pLASRct, peak strain rate during contractile phase; LATEF, left-atrial total ejection fraction; LATEV, left-atrial total ejection volume; LAV_max_, left-atrial volume during systole; LAV_min_, left-atrial volume during diastole; LAV_pre-A_, left-atrial volume before P-wave.

**Table 3:** Comparation of left-atrial (LA) strain parameters across HCM subjects with (G+) or without (G−) pathogenic (P)/likely pathogenic (LP) variants and hypertensive subjects with left-ventricular hypertrophy (LVH).

Variable	G+ HCM (*n* = 12)	G- HCM (*n* = 31)	HTN (*n* = 43)	*p* G+ vs. G−	*p* G+ vs. HTN	*p* G− vs. HTN
Age (years)	41 ± 11.45	54.3 ± 15.2	51.4 ± 13.1	0.009	0.015	0.38
Gender (male) (%)	12 (100%)	21 (67.7%)	30 (69.6%)	0.04	0.056	0.4
IVS (mm)	19.17 ± 5.13	20.45 ± 4.33	16.2 ± 2.6	0.24	0.008	<0.001
Posterior wall (mm)	13.27 ± 3.38	15.03 ± 3.73	14.2 ± 1.4	0.18	0.15	0.18
Indexed LV mass (g/m^2^)	163 ± 45.35	140.42 ± 43.35	92 ± 30.9	0.14	<0.001	<0.001
LVEF (%)	55 ± 14.46	55.1 ± 10.85	55 ± 9.2	0.14	0.98	0.96
LA diameter (mm)	43.2 ± 2.31	41.9 ± 7.16	40 ± 6.1	0.33	0.08	0.22
Volumetric Variables						
LAV_max_ (mL/m^2^)	33.16 ± 3.07	32.84 ± 3	29.89 ± 17.3	0.11	0.52	0.35
LAV_min_ (mL/m^2^)	15.16 ± 1.46	14.64 ± 1.64	16 ± 5	0.32	0.57	0.15
LAV_pre-A_ (mL/m^2^)	23 ± 2.4	25 ± 1.2	21 ± 1.4	0.001	0.001	<0.001
LATEF (%)	54.7 ± 15.3	56 ± 17.2	61 ± 8.9	0.82	0.07	0.10
LAEI (%)	167 ± 18.11	169 ± 25	191 ± 89	0.8	0.36	0.18
LAPEF (%)	26.2 ± 2.65	24.22 ± 5.74	26 ± 9.1	0.45	0.94	0.34
LAAEF (%)	44 ± 3.55	43.29 ± 1.98	47 ± 7.8	0.41	0.2	0.051
LAEF (kdyne)	11.1 ± 4.4	14 ± 3.1	13 ± 1.2	0.053	0.17	0.096
Deformation Variables						
Reservoir Function						
LASr (%)	24.7 ± 1.92	25 ± 2.7	32 ± 9.7	0.47	<0.001	<0.001
pLASRr (s^−1^)	0.93 ± 0.25	1.11 ± 0.28	1.9 ± 0.4	0.059	<0.001	<0.001
LASI	1.7 ± 0.2	1.8 ± 0.5	0.35 ± 0.2	0.22	<0.001	<0.001
Conduit Function						
LAScd (%)	17.82 ± 1.98	18.4 ± 2.91	22 ± 1.3	0.17	<0.001	<0.001
pLASRcd (s^−1^)	−0.42 ± 0.44	−0.7 ± 1.2	−1.2 ± 1.3	0.056	<0.001	<0.001
Contractile Function						
LASct (%)	10.3 ± 1.9	11.4 ± 2.37	12 ± 2.5	0.003	0.034	0.3
pLASRct (s^−1^)	−1.15 ± 0.4	−1.5 ± 0.23	−1.5 ± 1.3	<0.001	0.02	0.9

IVS, interventricular septum; LV, left ventricular; LAEF, left-atrial ejection force; LASI, left-atrial stiffness index; LAAEF, left-atrial active ejection fraction; LAEI, left-atrial ejection index; LAPEF, left-atrial passive ejection fraction; LAScd, left-atrial strain during conduit phase; LASct, left-atrial strain during contractile phase; LASr, left-atrial strain during reservoir phase; pLASRr, peak strain rate during reservoir phase; pLASRcd, peak strain rate during conduit phase; pLASRct, peak strain rate during contractile phase; LATEF, left-atrial total ejection fraction; LAV_max_, left-atrial volume during systole; LAV_min_, left-atrial volume during diastole; LAV_pre-A_, left-atrial volume before P-wave; LVEF, left-ventricular ejection fraction.

## Data Availability

Data available upon request.

## Supplementary Materials

Supplementary materials can be found at https://www.mdpi.com/article/10.3390/diagnostics11050814/s1.
