# Supplementary material for: The Role of Left-Atrial Mechanics Assessed by Two-Dimensional Speckle-Tracking Echocardiography to Differentiate Hypertrophic Cardiomyopathy from Hypertensive Left-Ventricular Hypertrophy
Source: Diagnostics (Basel). 2021 Apr 30;11(5):814. doi: 10.3390/diagnostics11050814 (PMC8145163; doi:10.3390/diagnostics11050814)
Supplement: Supplementary file 1 [file diagnostics-11-00814-s001.zip › diagnostics-1165212-supplementary.pdf]

Supplementary Table S1 Left atrial parameters analysed and formulas

Variable	Abbreviation	Formula
LA volumetric analysis and LA ejection force		
**LA reservoir**		
Left atrial total ejection volume	LATEV (ml)	LAV_{max}-LAV_{min}
Left atrial total ejection fraction	LATEF (%)	LATEV/LAV_{max}
Left atrial ejection index	LAEI (%)	LATEV/LAV_{min}
**LA conduit function**		
Left atrial passive ejection volume	LAPEV (ml)	LAVmax -LAV_{pre-A}
Left atrial passive ejection fraction	LAPEF (%)	LAPEV/ LAV_{max}
**LA pump function**		
Left atrial active ejection volume	LAAEV (ml)	LAV_{pre-A}-LAV_{min}
Left atrial active ejection fraction	LAAEF (%)	LAAEV/LAV_{pre-A}
Left atrial ejection force	LAEF (kdyne)	0.5×1.06×mitral annulus area× (peak A velocity^2)
LA deformation analysis		
**Reservoir function**		
Left atrial strain during reservoir phase	LASr (%)	$S_{mitral\ valve\ opening}$ -S_{LVED}
Peak strain rate during reservoir phase	pLASRr (sec^{-1})	positive peak strain rate during reservoir phase
Left atrial stiffness index	LASI	E/e'm/LASr
**Conduit function**		
Left atrial strain during conduit phase	LAScd (%)	S_{LVED} -$S_{atrial\ contraction}$
Peak strain rate during conduit phase	pLASRcd (sec^{-1})	negative peak strain rate during conduit phase
**Contractile function**		
Left atrial strain during contractile phase	LASct (%)	$S_{atrial\ contraction}$-$S_{mitral\ valve\ opening}$
Peak strain rate during contractile phase	pLASRct (sec^{-1})	negative peak strain rate during contractile phase

A atrial velocity of mitral flow; e'm average of early diastolic velocity of lateral and mitral annulus; LAVmax left atrial volume during systole; LAVmin left atrial volume during diastole; LAVpre-A left atrial volume before P-wave; LVED left ventricle end-diastole.

Supplementary Table S2 Intra and inter-observer reliability for reservoir, conduit and contractile left atrial strain and strain rate

Parameter	Intra-observer 1 ICC	Intra-observer 1 95% CI	Intra-observer 2 ICC	Intra-observer 2 95% CI	Inter-observer ICC	Inter-observer 95% CI
Reservoir function						
LASr (%)	0.9	0.8-0.96	0.92	0.8-0.99	0.84	0.7-0.94
pLASRr (sec^{-1})	0.82	0.71-0.93	0.86	0.9-0.99	0.92	0.8-0.96
Conduit function						
LAScd (%)	0.78	0.81-0.9	0.9	0.82-0.99	0.8	0.7-0.88
pLASRcd(sec^{-1})	0.82	0.7-0.89	0.89	0.8-0.93	0.9	0.82-0.96
Contractile function						
LASct (%)	0.79	0.7-0.88	0.94	0.9-0.99	0.86	0.8-0.9
pLASRct (sec^{-1})	0.86	0.8-.092	0.89	0.8-0.92	0.71	0.7-0.9

CI confidence interval; ICC intraclass coefficient correlation; LASct left atrial strain during contractile phase; LASr left atrial strain during reservoir phase; pLASRr peak strain rate during reservoir phase; pLASRcd peak strain rate during conduit phase;pLASTct peak strain rate during contractile phase.

Supplementary Table S3 Association of two-dimensional echocardiography characteristics with LVH due to hypertrophic cardiomyopathy after adjustment for age and sex in all subjects with LVH (n=86)

Variables	Univariate analysis		Multivariate analysis	
	Odds ratio (95% CI)	p	Odds ratio (95% CI)	p
IVS (mm)	1.39 (1.05-1.43)	<0.001	1 (0.6-1.6)	0.051
E/e's	1.27 (0.88-0.98)	0.03	0.5 (0.2-1.4)	0.07
E/e'l	0.69 (0.42-0.86)	0.035	0.6 (0.1-1.4)	0.08
LAV_{max} (ml/m^2)	1.02 (1.05-1.73)	0.001	0.8 (0.2-1)	0.9
LASr (%)	0.11 (0.9-1.3)	0.058		
pLASRr (sec^{-1})	0.45 (0.5-0.97)	0.04	0.1 (0.1-0.9)	0.81
LASr/IVS	0.22 (0.91-1.06)	0.06		
pLASRr/IVS	0.98 (0.97-1.1)	0.05		
LASI	0.3 (0.1-0.9)	0.04	0.2 (0.1-0.95)	0.45
LAScd (%)	0.99 (0.96-1.04)	0.06		
pLASRcd (sec^{-1})	1.04 (1.01-1.31)	0.021	0.6 (1.3-2.1)	0.06
LAScd/IVS	1.8 (1.1-1.47)	0.02	0.5 (1.1-2.3)	0.05
pLAScd/IVS	1.1 (0.9-1.4)	0.09		
LASct (%)	1.2 (0.8-0.92)	0.01	1 (0.7-1.2)	0.05
pLASRct (sec^{-1})	1.1 (0.7-0.99)	0.04	1.1 (0.6-1.3)	0.06
LASct/IVS	1.8 (0.86-0.97)	<0.001	1.4 (0.7-0.9)	0.02
pLASRct/IVS	1.6 (0.8-0.99)	0.001	1.2 (0.7-0.98)	0.03

CI confidence interval; e'l early diastolic velocity of lateral annulus; e's early diastolic velocity of mitral annulus; IVS interventricular septum; LAScd left atrial strain during conduit phase; LASct left atrial strain during contractile phase; LASI left atrial stiffness index; LASr left atrial strain during reservoir phase; LAVmax left atrial volume during systole; LVH left ventricular hypertrophy; pLASRcd peak strain rate during conduit phase; pLASTct peak strain rate during contractile phase; pLASRr peak strain rate during reservoir phase.
